# Hospital-Acquired Pneumonia among Inpatients via the Emergency Department: A Propensity-Score Matched Analysis

**DOI:** 10.3390/ijerph15061178

**Published:** 2018-06-05

**Authors:** Jin-young Min, Hye-Jin Kim, Chungsik Yoon, Kiyoung Lee, Myoungsouk Yeo, Kyoung-bok Min

**Affiliations:** 1 Institute of Health and Environment, Seoul National University Graduate School of Public Health, 1 Gwanak-ro, Gwanak-gu, Seoul 08826, Korea; yaemin00@snu.ac.kr (J.-y.M.); csyoon@snu.ac.kr (C.Y.); cleanair@snu.ac.kr (K.L.); 2 Department of Preventive Medicine, College of Medicine, Seoul National University, 103 Daehak-ro, Jongno-gu, Seoul 03080, Korea; okimhj@snu.ac.kr; 3 Department of Environmental Health Sciences, Seoul National University Graduate School of Public Health, 1 Gwanak-ro, Gwanak-gu, Seoul 08826, Korea; 4 Department of Architecture and Architectural Engineering, College of Engineering, Seoul National University, 1 Gwanak-ro, Gwanak-gu, Seoul 08826, Korea; msyeo@snu.ac.kr

**Keywords:** hospital-acquired pneumonia, emergency department, inpatient, claim data, South Korea

## Abstract

*Background:* Hospital-acquired pneumonia (HAP) is an inflammatory condition of the lung that develops at least 48–72 h after admission. HAP is contracted by both intensive care unit (ICU) and non-ICU patients, but no studies have examined the risk of HAP in patients admitted to the emergency department (ED). This study investigated the risk of developing HAP in ED patients and compared the occurrence of HAP 3–10 days after the first day of hospitalization in patients hospitalized via ED with those hospitalized via outpatient clinics. *Methods:* We analyzed the 2010 National Inpatient Sample data collected by the Health Insurance Review and Assessment Service in South Korea. After propensity score matching for age, sex, residential area, hospital, and diseases, 153,130 inpatients (76,565 admitted via ED and 76,565 admitted via outpatient clinics) were included in the analysis. The diagnosis of pneumonia was based on the International Classification of Diseases and Related Health Problems 10th Revision (Pneumonia, all (J12–J18); Pneumonia, bacterial (J13–J15); Pneumonia, non-bacterial (J12, J16, J17); and Pneumonia, unspecified (J18)). *Results:* The percentage of newly diagnosed cases of pneumonia in inpatients admitted via ED was significantly higher than that in inpatients admitted via outpatient clinics. After propensity score matching for baseline characteristics, the likelihood of developing pneumonia (excluding the category of ‘Pneumonia, non-bacterial’) in inpatients hospitalized via ED was significantly increased by 1.33–1.97-fold. The cumulative incidence of pneumonia was also significantly higher in patients admitted via ED than in those hospitalized via outpatient clinics. *Conclusions:* ED visits may be an important risk factor for the development of HAP.

## 1. Introduction

Hospital-acquired pneumonia (HAP) is an inflammatory condition of the lung parenchyma that develops at least 48–72 h after admission [1]. HAP is the second most common cause of nosocomial infection after urinary tract infections [2]. HAP has a global incidence of 5–20 cases per 1000 hospital admissions and has important clinical and financial consequences [2,3]. HAP is of particular concern in intensive care unit (ICU) patients, many of whom are mechanically ventilated [1]. It has been previously reported that HAP accounts for nearly 25% of all infections in ICU patients [4]. The incidence of HAP is projected to increase by 6-fold to 20-fold in mechanically ventilated patients [5]. However, there is growing concern regarding the increasing incidence of HAP in non-ICU patients. The available data indicate that the incidence of HAP is in the range of 2.8–6.1 cases per 1000 non-ICU patients, such as patients in general, medical, and surgical wards [6,7,8,9]. HAP in non-ICU patients is associated with elevated morbidity and mortality rates and an increased length of hospital stay [8,9].

Emergency department (ED) is a challenging environment to control infectious diseases. ED is often overcrowded and ED patients are in close proximity to undifferentiated patients and patients with potentially infectious body fluids, which ultimately facilitates the transmission of infectious pathogens from person to person during medical treatment [10]. Given that ED is a gateway to the hospital and exhibits a high risk of infectious disease transmission, the risk of developing HAP during an ED visit may not differ from the risk of developing HAP outside ICU. However, only one study has examined the risk of developing HAP in ED, which focused on surgical patients with acute abdomen or trauma [11]. In this study, we investigated the risk of developing HAP in ED. We compared the incidence of pneumonia 3–10 days after hospitalization in patients hospitalized via ED and those hospitalized via outpatient clinics. The propensity score matching method was applied to balance the potential risk of HAP in the two inpatient groups.

## 2. Materials and Methods

South Korea has a national health insurance system that covers 98% of the population. The Health Insurance Review and Assessment Service (HIRA) collects the claim data submitted by the healthcare providers to review reimbursement coverage/non-coverage. These data apply to approximately 90% of the population per year. Based on this vast amount of original claim data, HIRA developed the Patient Samples data set [called as the HIRA-National Inpatient Sample (NIS)]. HIRA-NIS (serial number: HIRA-NIS-2010-0068) adopted a stratified randomized sample extraction method, which passed the required validity test [12]. In this study, we used the 2010 HIRA-NIS, which is a comprehensive inpatient data set that includes the data of 700,000 inpatients (13% of the total inpatients) and approximately 400,000 outpatients (1% of the total outpatients) per year.

From the 2010 HIRA-NIS, we initially selected 605,856 patients (age ≥ 20 years). Among them, we excluded 92,574 patients as they were: patients who were hospitalized after 20th December (*n* = 54,519); patients who had a diagnosis of pneumonia on the day of hospitalization (*n* = 18,386); patients who developed pneumonia within 2 days of hospitalization (*n* = 519); and patients who were admitted to ICU after hospitalization (*n* = 19,150). The remaining 513,282 patients were eligible for subsequent analysis.

For patients who were hospitalized more than once in 2010, we only included the first hospitalization. We followed up the occurrence of pneumonia in the applicable inpatients 3–10 days after the first day of hospitalization.

**Figure 1:**
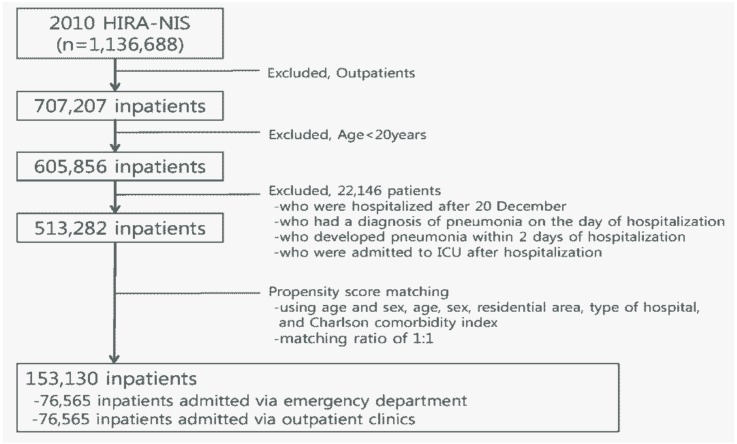
Flow chart of the study population.

We conducted propensity score matching using age, sex, residential area, type of hospital, and Charlson comorbidity index (CCI) with a matching ratio of 1:1. Subsequently, 76,565 inpatients admitted via ED were matched with 76,565 inpatients admitted via outpatient clinics, with a total of 153,130 inpatients finally being included in this study (Figure 1).

The baseline characteristics included age (20–29, 30–39, 40–49, 50–59, 60–69, 70–79, or ≥80), sex (male or female), place of treatment (urban or rural), type of hospital (university hospital or clinics/hospital), and CCI with three categories (0, 1, or ≥2).

As an outcome variable, the diagnosis of pneumonia was defined by the International Classification of Disease and Related Health Problems 10th Revision (ICD-10, available at http://apps.who.int/classifications/icd10) and was classified into four categories: Pneumonia, all (J12–J18); Pneumonia, bacterial (J13–J15); Pneumonia, non-bacterial (J12, J16, J17); and Pneumonia, unspecified (J18).

In the statistical analysis, we compared the baseline characteristics between the matched inpatient groups (inpatients admitted via ED versus those admitted via outpatient clinics) using the Chi-square test. The Cox proportional hazards regression analysis was performed to estimate the probability of developing pneumonia in inpatients admitted via ED or via outpatient clinics 3–10 days after the first day of hospitalization. The regression model included all baseline characteristics as confounding variables, which subsequently computed the hazard ratio (HR) and 95% confidence interval (CI). In addition, the cumulative incidence plots of pneumonia per 1000 inpatients were generated for the four pneumonia categories. All statistical analyses were performed using SAS 9.4 (SAS Institute Inc., Cary, NC, USA), and the statistical significance level was set at α = 0.05.

## 3. Results

After propensity score matching, there were no differences in characteristics between the two inpatient groups (all *p*-values were < 0.05; Supplementary Table S1).

**Figure 2:**
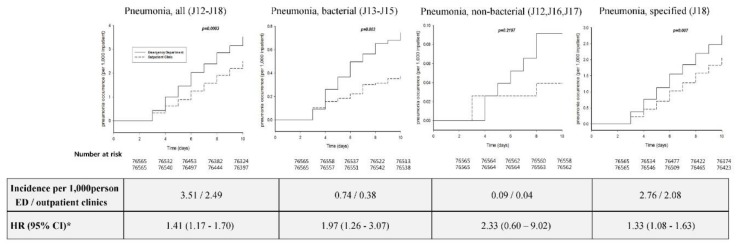
Cumulative incidence plots, number of cases, and HR for pneumonia occurrence. The solid lines indicate inpatients admitted via ED, while the dotted lines indicate inpatients admitted via outpatient clinics.* HR was adjusted by age, sex, residential area, type of hospital, and CCI. HR, hazard ratio; ED, emergency department; CCI, Charlson comorbidity index.

Figure 2 shows the percentage of cases and HR for the occurrence of pneumonia in inpatients 3–10 days after the first day of hospitalization. Apart from non-bacterial pneumonia, the percentage of newly diagnosed cases of pneumonia in inpatients admitted via ED was significantly higher than that in inpatients admitted via outpatient clinics. After adjusting for confounding variables, the HRs (95% CIs) in inpatients hospitalized via ED were as follows: 1.41 (1.17–1.70) for Pneumonia, all; 1.97 (1.26–3.07) for Pneumonia, bacterial; and 1.33 (1.08–1.63) for Pneumonia, unspecified. Cumulative incidence plots indicated that inpatients admitted via ED had a higher incidence of pneumonia than those admitted via outpatient clinics: 3.51 versus 2.49 cases per 1000 patients for Pneumonia, all (*p* < 0.0001); 0.74 versus 0.38 cases per 1000 patients for Pneumonia, bacterial (*p* = 0.0498); and 2.76 versus 2.08 cases per 1000 patients for Pneumonia, unspecified (*p* < 0.0001). In contrast, there was no significant difference in the HR and cumulative incidence of pneumonia for Pneumonia, non-bacterial. 

## 4. Discussion

We found that ED visits were associated with the development of pneumonia in non-ICU hospitalized patients. After propensity score matching using the demographic and clinical characteristics of the inpatients, the likelihood of developing pneumonia (excluding Pneumonia, non-bacterial) was significantly increased by 1.33–1.97-fold. The cumulative incidence of all pneumonia (ICD-10: J12–J18) was 3.51 cases per 1000 patients 3–10 days after the first day of hospitalization. Although further studies are needed to confirm our results, our data suggest that ED visits may be a risk factor for the development of HAP.

To the best of our knowledge, no studies have examined the risk of pneumonia in inpatients hospitalized via ED relative to those hospitalized via outpatient clinics. However, a recent study reported the risk of HAP in surgical patients admitted to ED [11]: of 4961 surgical patients with acute abdomen or trauma, 90 (1.8%) were diagnosed with pneumonia more than 48 h after admission. Verified or suspected aspiration, immobilization, and chronic pulmonary obstructive disease/asthma were significantly increased in these surgical patients with HAP. It is impossible to directly compare these findings with our own because of the differences in study design and patients enrolled. However, these data emphasize a potential correlation between the development of HAP and ED visits, which is consistent with our findings.

The mechanism underlying the development of ED-associated HAP is unclear but it is likely multifactorial. The most feasible risk factor is the mechanical ventilation performed in ED. Indeed, many ED patients have trauma or other critical illnesses and require mechanical ventilation or an admission to ICU through ED [10]. We excluded patients hospitalized in ICU after ED visits or outpatient clinic visits to minimize the possibility of ventilation-associated HAP as much as possible. In ED, the causative pathway of HAP may involve aspiration of the upper respiratory tract and/or the inhalation of aqueous or airborne aerosols carrying pneumonia pathogens, in which causes HAP in non-ICU patients [13]. However, previous studies have noted the occurrence of HAP in non-ICU patients not receiving mechanical ventilation [6,7,8,9]. The authors considered patients’ health status (i.e., advanced age, comorbidities, malnutrition, and/or depression of consciousness) and therapeutic procedures (i.e., the use of invasive thoracic devices and equipment, nasogastric tubes, and immunosuppressive treatment) as risk factors for its occurrence [6,7,8,9]. The causes of HAP in patients hospitalized via ED do not differ. ED patients are more likely to have a poor health status or require invasive medical procedures [10], which are known risk factors for HAP [13]. As ED is conductive to the transmission of infectious diseases, many types of infection are transmitted through healthcare devices, environment (i.e., air), or transfer of microorganisms between healthcare workers and patients [10]. Further studies are needed to identify the potential risk factors for HAP in ED. 

## 5. Conclusions

In conclusion, inpatients hospitalized via ED were at a higher risk of HAP than those hospitalized via outpatient clinics. Our results were based on a large, representative sample of the inpatient data gathered in South Korea. However, the HIRA-NIS has inherent limitations because of the lack of information on patients, hospitals, hospital activities (i.e., paraclinic examinations and treatment procedures), and the accuracy and validity of diagnostic codes. Thus, we cannot completely exclude the possibility that bias may have distorted the results. Thus, further studies with more detailed data are needed, while preventive strategies for ED patients should be developed to improve patient safety with regard to infectious diseases, such as HAP.

## Supplementary Materials

The following are available online at www.mdpi.com/1660-4601/15/6/1178/s1.
