# Supplementary material for: Hospital-Acquired Pneumonia among Inpatients via the Emergency Department: A Propensity-Score Matched Analysis
Source: Int J Environ Res Public Health. 2018 Jun 5;15(6):1178. doi: 10.3390/ijerph15061178 (PMC6024900; doi:10.3390/ijerph15061178)
Supplement: Supplementary file 1 [file ijerph-15-01178-s001.pdf]

Supplement Table 1. Baseline characteristics of inpatients after propensity score matching (n = 153,130).

Characteristics	no. (%) of Inpatients				p-Value
	Hospitalization after ED Visits (n = 76,565)		Hospitalization after Outpatient Visits (n = 76,565)		
Age (years)					
20–29	9842	(50)	9842	(50)	1.000
30–39	12,196	(50)	12,198	(50)	
40—49	12,926	(50)	12,926	(50)	
50–59	13,433	(50)	13,433	(50)	
60–69	11,926	(50)	11,926	(50)	
70–79	11,076	(50)	11,076	(50)	
≥80	5166	(50)	5164	(50)	
Gender					
Male	36,774	(50)	36,776	(50)	0.992
Female	39,791	(50)	39,789	(50)	
Residential area					
Urban area	37,973	(50)	37,973	(50)	1.000
Rural area	38,592	(50)	38,592	(50)	
Type of hospitals					
University hospitals	24,650	(50)	24,650	(50)	1.000
Clinics/hospitals	51,915	(50)	51,915	(50)	
CCI					
0	28,568	(50)	28,568	(50)	1.000
1	22,254	(50)	22,252	(50)	
≥2	25,743	(50)	25,745	(50)	

CCI, Charlson Comorbidity Index.
